# Development of Novel PANoptosis‐Related Gene Signatures to Predict the Prognosis of Patients With Stomach Adenocarcinoma

**DOI:** 10.1155/humu/5319444

**Published:** 2025-12-21

**Authors:** Xin Wu, Linde Sun, Peifa Liu, Qiong Wang, Yu Wang

**Affiliations:** ^1^ Department of General Surgical Medicine, The First Medical Center of PLA Hospital, Beijing, China; ^2^ Pathology Department, The First Medical Center of PLA Hospital, Beijing, China

**Keywords:** molecular subtype, PANoptosis, prognostic signature, RiskScore, stomach adenocarcinoma, tumor microenvironment

## Abstract

**Background:**

PANoptosis, as an inflammatory programmed cell death, is involved in tumor development. This study set out to discover novel PANoptosis‐correlated prognostic signatures in stomach adenocarcinoma (STAD), a prevalent malignancy of the digestive system.

**Methods:**

STAD samples were derived from a public database, and PANoptosis‐related genes (PRGs) were acquired from existing reports. Prognosis‐related PRGs were screened by univariate Cox regression analysis. Molecular subtypes of STAD were identified by the “ConsensusClusterPlus” package. The “Limma” package was employed to filter differentially expressed genes (DEGs) between different subtypes. PANoptosis‐related prognostic signatures in STAD were identified to establish the RiskScore model. The RiskScore and some of the clinical features were integrated to establish a nomogram. Immune cell infiltration and TIDE score in different risk groups were compared. Correlation between immune checkpoint genes, drug sensitivity, and RiskScore was analyzed by the Spearman method. The biological function of PANoptosis‐related signature genes in STAD was preliminarily explored by in vitro cell experiments.

**Results:**

Based on 18 prognosis‐related PRGs, two molecular subtypes of STAD were recognized, and the C1 subtype showed a lower overall survival (OS) rate than the C2 subtype. Further, three PANoptosis‐related signature genes (*APOD*, *GPC3*, and *SERPINE1*) were determined to establish a RiskScore model that could accurately assess the prognostic outcomes for STAD patients. Then, by integrating RiskScore with clinical features, a nomogram was established. The high‐risk group had higher immune cell infiltration and TIDE score and lower OS rate than those with a low risk. RiskScore was positively correlated with nine immune checkpoint genes. Besides, we screened 23 drugs that significantly correlated with RiskScore. In vitro cell experiments showed that the mRNA and protein levels of *APOD*, *GPC3*, and *SERPINE1* were upregulated in the STAD cell line and that *APOD* knockout significantly reduced cancer cell proliferation, migration, and invasion levels and increased the apoptotic capacity of the STAD cell line.

**Conclusion:**

This study established a PANoptosis‐related RiskScore model for assessing STAD patient prognosis, which could contribute to the personalized treatment of STAD.

## 1. Introduction

Gastric cancer (GC) is a frequent cause of carcinoma‐relevant death around the world [1]. Stomach adenocarcinoma (STAD) belongs to a histological type of GC and accounts for 95% of gastric malignancy [2, 3]. Statistics showed that about 951,600 new cases and about 723,100 deaths of STAD occur every year globally [4]. STAD is characterized by strong invasiveness and high molecular heterogeneity, which affects patient prognosis and accurate treatment [5]. Nowadays, the 5‐year survival of STAD patients with Stages IA and IB treated with surgery can reach 60%–80% [6]. However, owing to unobvious clinical symptoms at the early stage, most patients with STAD have developed to an advanced stage or even distant metastases when detected [7]. The patients with advanced or metastatic STAD, who miss the optimal time for treatment, have a 5‐year survival probability of no more than 30% [8]. In addition, despite revolutionary advances in the treatment of STAD through chemotherapy, targeted therapy, and immunotherapy, the patient′s prognosis remains unsatisfactory due to tumor relapse, drug resistance, or low sensitivity [9]. Therefore, identifying reliable and accurate signatures can improve the survival of STAD by contributing to early diagnosis, prognostic evaluation, or serving as therapeutic targets.

Programmed cell death (PCD) has critical functions in the maintenance of cell status [10, 11]. PANoptosis is a novel proposed pattern of inflammatory PCD that is promiscuously implicated in the development and progression of numerous cancers, including hepatocellular carcinoma [12], breast cancer [13], pancreatic adenocarcinoma [14], and GC [15]. PANoptosis has the main characteristics of necroptosis, pyroptosis, and apoptosis [16]. PANoptosis is regulated by the PANoptosome complex, but it could not be separately interpreted by any of the three PCD pathways [17]. Apoptosis is a procedure to form an apoptotic body and avoid an inflammatory response. Pyroptosis and necroptosis can destroy the cell membrane and secrete inflammatory factors to entice an inflammatory response [18]. An increasing number of researches have highlighted the crosstalk and coordination of pyroptosis, apoptosis, and necroptosis [19]. A study has confirmed the role of PANoptosis in GC. For instance, Qing et al. revealed the immunological characteristics of PANoptosis‐related genes (PRGs) in GC by machine learning algorithms and indicated that the PANoptosis signatures could be applied as a promising risk stratification tool for GC patients [20]. Lin et al. demonstrated that YBX1 could increase the tumor resistance to oxaliplatin in GC via suppressing PANoptosis [21]. Nonetheless, the prognostic significance of PRGs in STAD has not been explored further. In consequence, the specific roles of PANoptosis in STAD need to be further studied to mine novel prognostic signatures and therapeutic targets.

Based on prognosis‐related PRGs, the present work classified the molecular subtypes of STAD. The overall survival (OS) rate, clinical features, and DEGs between different molecular subtypes were compared. Then, the PANoptosis‐related prognostic signatures in STAD were filtered to develop a RiskScore model and the robustness of which was validated. RiskScore and clinical features were integrated together to create a nomogram. Furthermore, immune cell infiltration and response to immunotherapy in different risk groups were assessed. The relationships among drug sensitivity, immune checkpoint genes, and RiskScore were also analyzed. Moreover, in vitro cell experiments were also conducted to preliminarily explore the biological function of PANoptosis‐related signature genes in STAD. We hope this study paves the way for improving prognosis and developing new treatment strategies for STAD.

## 2. Material and Methods

### 2.1. Data Sources and Preprocessing

The Cancer Genome Atlas (TCGA)‐STAD cohort in FPKM format was collected from the UCSC Xena (https://xena.ucsc.edu/) database. Then, samples without clinical follow‐up or with a survival time of ≤ 30 days were deleted. Gene symbol was converted from Ensembl, and the maximum expression value was taken with multiple gene symbols. The expression matrix was transformed into a format of TPM and log2‐converted. Eventually, 366 STAD samples and 32 para‐cancer control samples were obtained in the TCGA‐STAD cohort, which was utilized as a training set.

From the Gene Expression Omnibus (GEO) database, the expression and clinical data of the GSE62254 dataset (https://www.ncbi.nlm.nih.gov/geo/query/acc.cgi?acc=GSE62254) were obtained. The probes were mapped to genes according to the annotation information of the chip platform while removing the probes matching multiple genes. The mean expression value of the gene was taken when multiple probes matched one gene. At the same time, samples with a survival time of ≤ 30 days were deleted. In this way, a sum of 300 STAD samples were collected in the GSE62254 dataset that was applied as a validation set.

A total of 277 PRGs were acquired from a previous study [22].

### 2.2. Screening of Prognosis‐Related PRGs

Prognosis‐related PRGs in the TCGA‐STAD cohort were screened by applying univariate Cox regression analysis (*p* < 0.05). Then, the expressions of prognosis‐related PRGs in control and STAD samples were compared. The mutation frequency of prognosis‐related PRGs in STAD was evaluated and visualized by a waterfall plot using the “maftool” R package [23].

### 2.3. Identification of Molecular Subtypes of STAD

Using the prognosis‐related PRGs, the molecular subtypes of STAD were classified. Firstly, the enrichment score of prognosis‐related PRGs in the TCGA‐STAD cohort was calculated by ssGSEA with the “GSVA” R package [24]. Next, the STAD samples were clustered by consistency clustering with the “ConsensusClusterPlus” R package [25]. The “km” algorithm and “1‐Spearman correlation” were set as metric distances. A total of 500 bootstraps were performed, and each bootstrap comprised 80% patients of the training set and 20% patients of the validation set. The optimal classification was decided according to a consensus matrix with cluster number (*k*) = 2‐10. Further, the OS between different molecular subtypes was compared by the Kaplan–Meier (K‐M) curve analysis. The distribution of clinicopathological features was also analyzed in different molecular subtypes.

### 2.4. Functional Enrichment Analysis

Utilizing the “limma” R package [26], DEGs between the molecular subtypes were recognized under the threshold values of false discovery rates (FDR) < 0.05 and |log2foldchange(FC)| > log2(2). KEGG and GO enrichment analyses were conducted on these genes, applying the “clusterProfiler” R package [27].

### 2.5. Development and Verification of the RiskScore Model

The DEGs were subjected to univariate Cox regression analysis to screen the prognostic genes (*p* < 0.05). To reduce the gene numbers, LASSO regression analysis was conducted using the “glmnet” R package [28]. The optimal RiskScore model was formulated with 10‐fold cross‐validation and stepwise multivariate regression analysis based on all samples in the TCGA‐STAD cohort [29]:

RiskScore=∑βi∗ExPi,

where *β* is the coefficient of the gene in the Cox regression model and *i* is the expression value of the gene.

RiskScore was normalized by *z*‐score, and patients in the TCGA‐STAD cohort were separated into high‐ and low‐risk groups by a threshold value of 0. The OS rate between different risk groups was compared by the K‐M curve, accompanied by the log‐rank test. Receiver operating characteristic (ROC) was used for assessing the predictive performance of the RiskScore model by the “timeROC” R package [30]. In addition, the robustness of the RiskScore was verified in the GSE62254 dataset.

### 2.6. Development and Verification of Nomogram

Whether the RiskScore was independent of other clinicopathological characteristics, such as pathologic_T, pathologic_N, pathologic_M, stage, age, and gender, was assessed using univariate and multivariate Cox regression analysis on the TCGA‐STAD cohort. Next, a nomogram was created by integrating RiskScore and clinicopathological features. The calibration curve was utilized to evaluate the prediction accuracy of the nomogram. The clinical utility of the nomogram was analyzed according to the decision curve analysis (DCA).

### 2.7. Analysis of Immune Cell Infiltration and Immunotherapy Response

The correlation between tumor microenvironment (TME), RiskScore, and immune cell infiltration in different risk groups in the TCGA‐STAD cohort was evaluated. TIMER [31] and the MCP‐counter algorithm [32] were employed to assess the abundance of six immune cells and the infiltration levels of 10 immune cells, respectively.

The immunotherapy response of different risk groups was evaluated by the TIDE algorithm [33]. Meanwhile, the correlations between RiskScore, prognostic signatures, and immune checkpoint genes were analyzed.

### 2.8. Drug Sensitivity Analysis

Using the “oncoPredict” R package [34], the half maximum inhibitory concentration (IC_50_) value of each drug was predicted for the patients in the TCGA‐STAD cohort. Then, the relationship between RiskScore and drug sensitivity was analyzed to screen the drugs with significant differences (FDR < 0.05 and |cor| > 0.3).

### 2.9. Cell Lines and Cultivation

The human stomach epithelial cell line GES‐1 (C5068, RRID: CVCL_EQ22) and STAD cell line AGS (C5025, RRID: CVCL_0139) were purchased from BDBio Co., Ltd. (Hangzhou, China). Another STAD cell line, HGC‐27 (CL‐0107, RRID: CVCL_1279), was purchased from Wuhan Pricella Biotechnology Co., Ltd. All these cell lines were confirmed by short tandem repeat analysis. GES‐1 cell was cultured in RPMI‐1640 medium (C5068‐500, BDBio, China) containing 10% fetal bovine serum (FBS). AGS cell was cultured in F‐12K medium (C5025‐500, BDBio, China) encompassing 10% FBS. HGC‐27 cell was cultured in RPMI‐1640 medium (CM‐0107) containing 10% FBS and 1% penicillin–streptomycin. These cells were stored with 5% CO_2_ at 37°C.

Further, the small interfering (si) RNAs of *APOD* (si‐APOD#1: 5 ^′^‐AGGAGAATTTTGACGTGAATAAG‐3 ^′^; si‐APOD#2: 5 ^′^‐GGCCAACTACTCACTAATGGAAA‐3 ^′^) and small interfering negative control (si‐NC) were provided by Shanghai GenePharma Co., Ltd. for silencing the expression of *APOD* in STAD cells. Lipofectamine 3000 Transfection Reagent (L3000008, Invitrogen, Carlsbad, California, United States) was employed to transfect the STAD cells with si‐APOD.

### 2.10. Quantitative Reverse Transcription PCR (qRT‐PCR)

Following the manual, the total RNA was extracted from HGC‐27, GES‐1, and AGS cells using TRIzol reagent (T751379‐100ml, Aladdin, Shanghai, China), which was reverse‐transcribed into cDNA with the help of SuperRT cDNA Synthesis Kit (S665657‐100T, Aladdin, China). Next, the UltraBio SYBR Green qPCR Mix (S751587‐25ml, Aladdin, China) was applied for qRT‐PCR amplification under the CG‐05 PCR System (HealForce, Shanghai, China). The relative mRNA expressions of the interested genes were determined by the 2^−*Δ* *Δ*CT^ method [35]. The primer sequences were listed in Table 1, and *GAPDH* served as the housekeeping gene.

**Table 1:** The primer sequences of this study.

**Gene**	**Forward sequence (5** ^′^ **to 3** ^′^ **)**	**Reverse sequence (5** ^′^ **to 3** ^′^ **)**
*APOD*	CAACTACTCACTAATGGAAAACGG	CTTGGCAGGCTCTGTGAGGTTA
*GPC3*	CATTGGAGGCTCTGGTGATGGA	TTGTCCTTCGGAGTTGCCTGCT
*SERPINE1*	CTCATCAGCCACTGGAAAGGCA	GACTCGTGAAGTCAGCCTGAAAC
*GAPDH*	GTCTCCTCTGACTTCAACAGCG	ACCACCCTGTTGCTGTAGCCAA

### 2.11. Western Blot

The analysis of protein levels in cell cultures subjected to various conditions and treatments was carried out using Western blotting. In summary, total cellular protein was obtained through the use of RIPA lysis buffer (Servicebio, Wuhan, China), which included protease and phosphatase inhibitors. Following this, the concentration of the extracted protein was quantified via a BCA Protein Assay Kit (Servicebio). For the electrophoresis, 50 *μ*g of protein was applied to 10% SDS‐PAGE gels, which were subsequently transferred to polyvinylidene fluoride membranes. After a 1‐h blocking period with 5% skim milk powder, the membranes were incubated overnight at 4°C with primary antibodies (Abcam, Cambridge, United Kingdom) and then incubated for an additional hour at 25°C with the HRP‐conjugated secondary antibody (ab205718, 1/2000, Abcam). The primary antibodies used were as follows: *APOD* (ab108191, 1/1000), *GPC3* (ab174851, 1/1000), *SERPINE1* (ab222754, 1/1000), and GAPDH (ab128915, 1/10 000). GAPDH served as the loading control. Ultimately, to visualize the bands of the target protein, enhanced chemiluminescence reagents were employed, and ImageJ software was utilized to analyze their grayscale.

### 2.12. Cell Viability Test

CCK‐8 assay was performed to assess the cell viability of *APOD*‐silenced AGS and HGC‐27 cells. The cells after transfection with si‐APOD (5 × 10^3^ cells/well) were planted into a 96‐well plate. During culture at 24, 48, and 72 h, each well was added with 10 *μ*L CCK‐8 solution (CA1210, Solarbio, Beijing, China). After incubation for 30 min, under the BioTek Synergy H1 microplate reader (Agilent, Santa Clara, California, United States), the optical density at 450 nm was detected [36].

### 2.13. Cell Migration Assessment

The migration of *APOD*‐silenced AGS and HGC‐27 cells was detected by performing a wound healing experiment. The transfected AGS and HGC‐27 cells at a density of 5 × 10^5^ cells/well were seeded into the six‐well plate. Thereafter, a 10‐*μ*L pipette tip was applied to create artificial wounds. The representative images at 0 and 48 h were captured by the CKX53 inverted microscope (RRID: SCR_025025, Olympus, Tokyo, Japan), and the wound closure rate (%) was estimated [37].

### 2.14. Cell Invasion Assessment

Transwell experiment was used to evaluate the cell invasion of *APOD*‐silenced AGS and HGC‐27 cells. The transfected AGS and HGC‐27 cells (3 × 10^4^ cells/well) were seeded into the Transwell upper chamber (8 *μ*m pores, Corning, Inc., Corning, New York, United States) precoated with Matrigel in 200 *μ*L serum‐free medium, while the lower chamber was filled with 600 *μ*L complete medium without cells. After 48 h, the cells that invaded into the lower chamber were fixed with 4% paraformaldehyde and dyed with 0.1% crystal violet. The representative photographs were captured under the above inverted microscope, and the invaded cell number was counted [38].

### 2.15. Flow Cytometry

STAD cells that were transfected with either APOD‐specific siRNA or a control si‐NC were gathered, washed with PBS, and resuspended in 195 *μ*L of Annexin V‐FITC working solution (BD Biosciences, Franklin Lakes, New Jersey, United States) mixed with 5 *μ*L of propidium iodide (PI), following the provided guidelines. Afterward, a 10‐min incubation of the cells in the dark at room temperature was conducted, and flow cytometry was employed for analysis. The data were assessed using Lysis software (EPICS‐XL, Ramsey, Minnesota, United States).

### 2.16. Statistical Analysis

Data analysis was performed utilizing R software (Version 4.1.0) and GraphPad Prism (Version 8.0, RRID: SCR_002798). The experiments were conducted in triplicate. Two‐group differences were compared by *t*‐tests. One‐way or two‐way ANOVA was employed to compare the differences among three or more groups. Correlation analysis was performed applying the Spearman method. A *p* < 0.05 was defined as a significant difference.

## 3. Results

### 3.1. Screening of 18 Prognosis‐Related PRGs

By univariate Cox regression analysis, we obtained 18 prognosis‐related PRGs in STAD (*p* < 0.05), including three “protective” genes with hazard ratio (HR) < 1 and 15 “risk” genes with HR > 1 (Figure 1a). Then, comparison of the expressions of 18 prognosis‐related PRGs in STAD samples and control samples showed that, compared to control samples, *SNAI2*, *MAGED1*, *LY96*, *IRF1*, *IL1R1*, and *CHMP4C* were markedly upregulated, yet *PDK4*, *ELANE*, *BMX*, and *ARHGAP10* were notably downregulated in STAD samples (Figure 1b). Most of the prognosis‐related PRGs were positively correlated with other genes (Figure 1c). Furthermore, we assessed the mutation frequency of 18 prognosis‐related PRGs in STAD, finding that only seven of these genes exhibited a mutation frequency greater than 1%, such as *MAGED1*, *SNAI2*, *NTRK1*, and *NGF* (Figure 1d).

Figure 1Screening of prognosis‐correlated PRGs in STAD. (a) Univariate Cox regression result of 18 prognosis‐related PRGs. (b) The expressions of 18 prognosis‐related PRGs in STAD samples and control samples. (c) Correlation analysis between 18 prognosis‐related PRGs. (d) Mutation frequency of 18 prognosis‐related PRGs in STAD.  ^∗∗∗∗^ *p* < 0.0001,  ^∗∗∗^ *p* < 0.001,  ^∗∗^ *p* < 0.01, and  ^∗^ *p* < 0.05; ns represents not significant.

**Figure:**
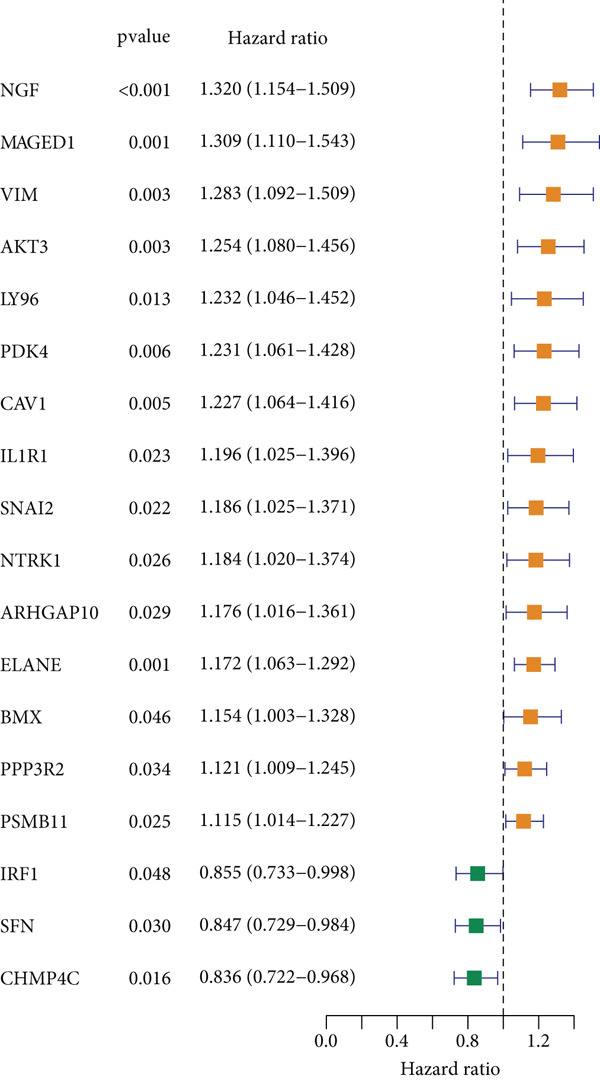
(a)

**Figure:**
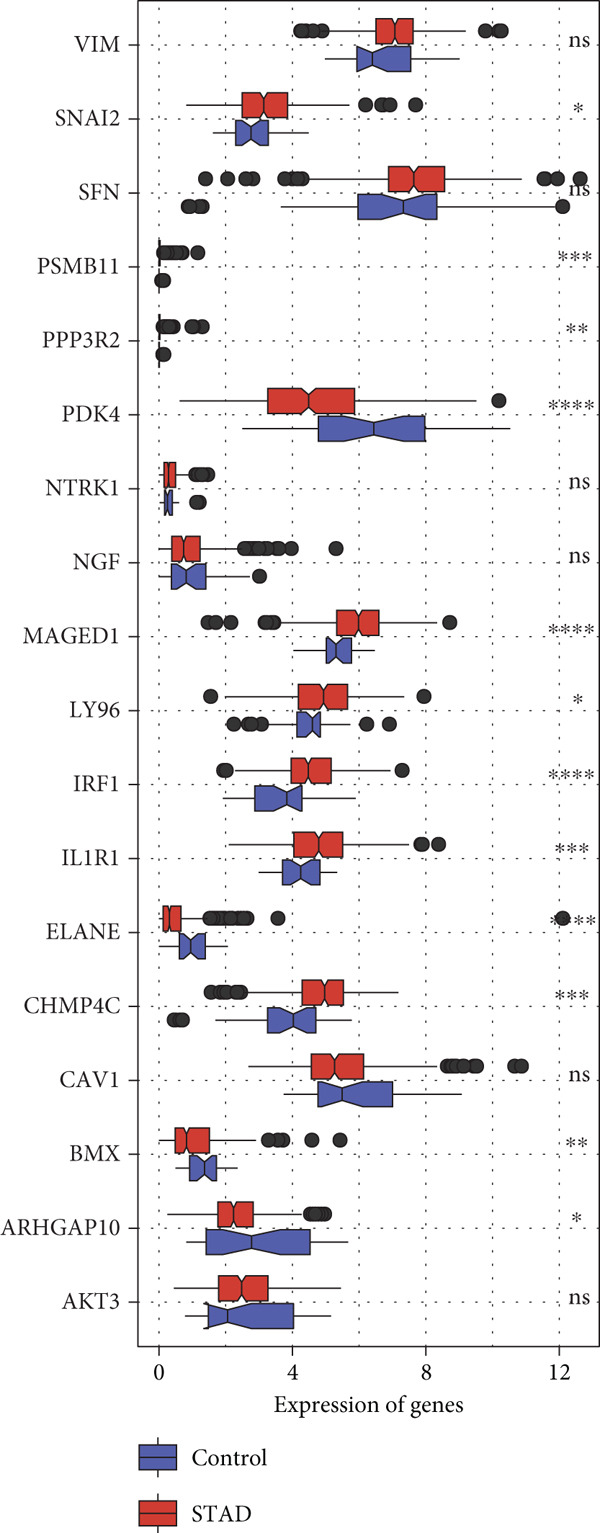
(b)

**Figure:**
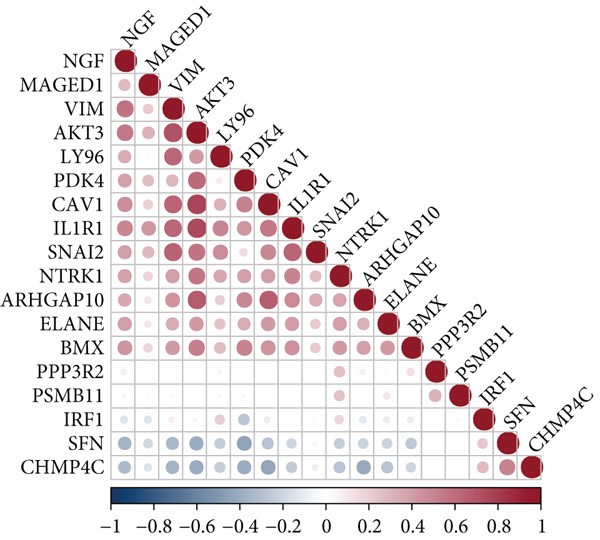
(c)

**Figure:**
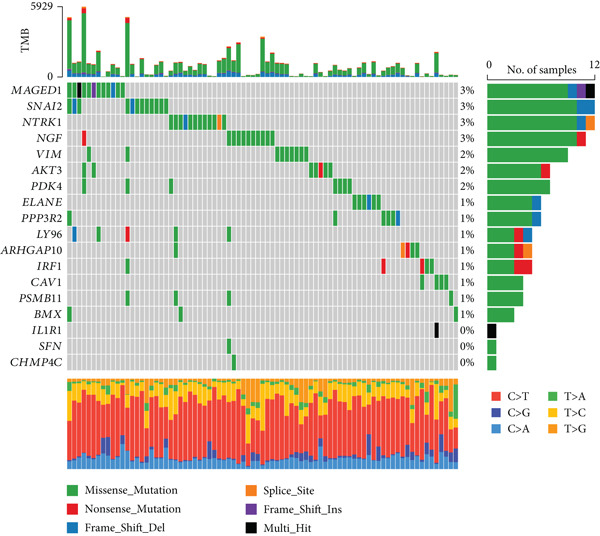
(d)

### 3.2. Two Molecular Subtypes of STAD Were Recognized Based on Prognosis‐Related PRGs

The enrichment score of 18 prognosis‐related PRGs was notably lower in STAD samples compared with control samples (Figure 2a). Further, consistency clustering analysis suggested that STAD patients were optimally classified into two clusters when the consensus matrix *k* = 2 (Figure 2b). The OS of the C1 subtype was markedly shorter than the C2 subtype (Figure 2c), which demonstrated that STAD patients of the C1 subtype exhibited a poorer prognosis. Additionally, we observed significant differences between C1 and C2 subtypes in stage, pathologic_T, and pathologic_N (Figure 2d), suggesting that these clinical features may be associated with subtype stratification.

Figure 2Identification of molecular subtypes for STAD. (a) ssGSEA score of STAD samples and control samples;  ^∗∗∗∗^ *p* < 0.0001. (b) Consistency clustering heat map of the TCGA‐STAD dataset. (c) Kaplan–Meier (K‐M) curve of overall survival (OS) between two molecular subtypes. (d) The chi‐squared test was used to assess significant differences in various clinical characteristics (including gender, age, stage, pathologic_T, pathologic_N, and pathologic_M) between the C1 and C2 subtypes.

**Figure:**
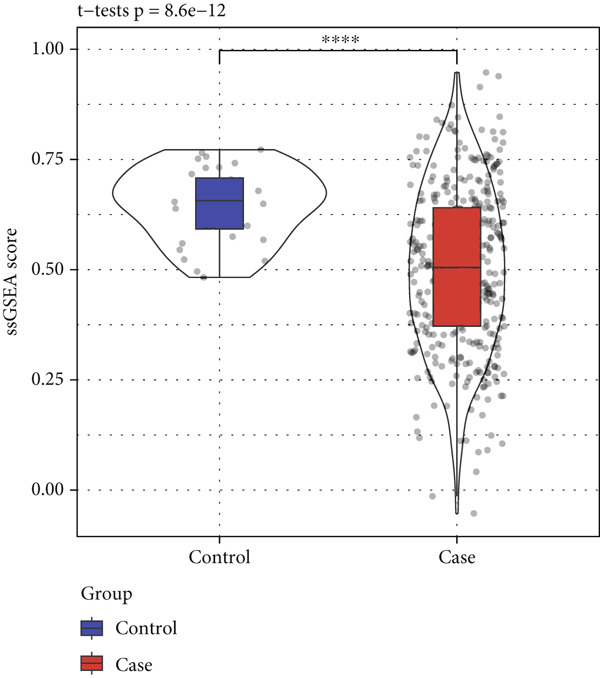
(a)

**Figure:**
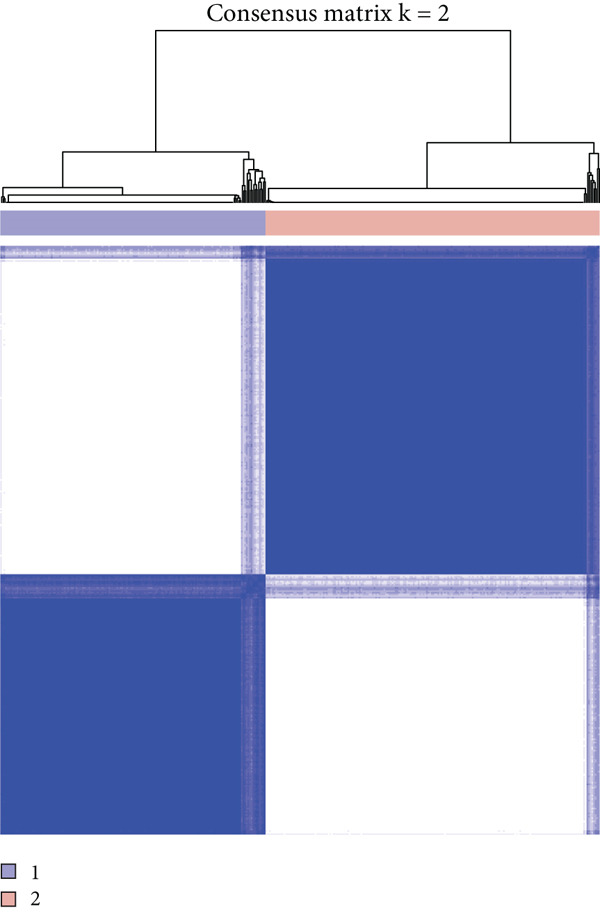
(b)

**Figure:**
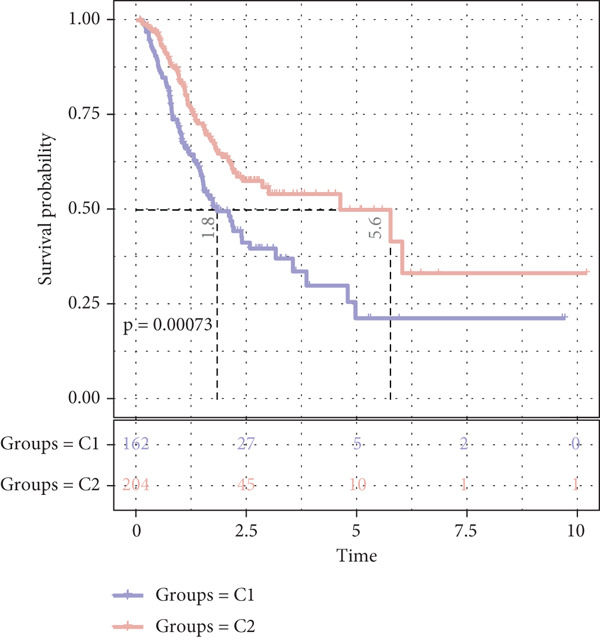
(c)

**Figure:**
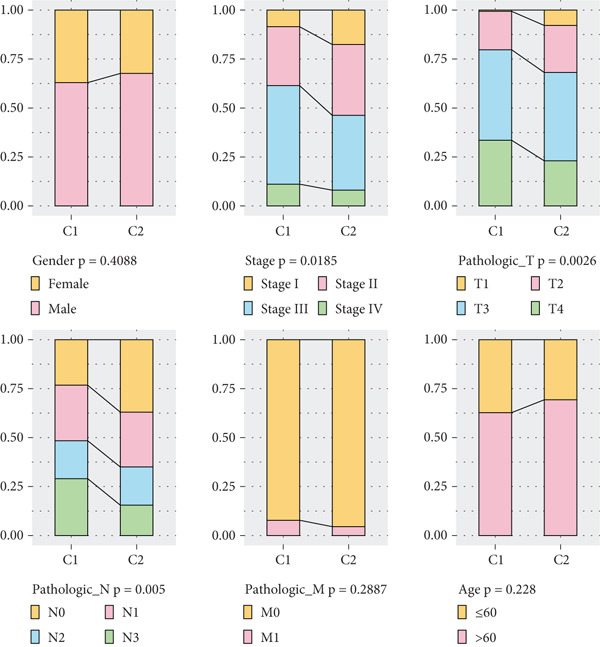
(d)

### 3.3. Functional Enrichment Analysis on the DEGs Between Two Molecular Subtypes

Firstly, we acquired 854 DEGs between C1 and C2 subtypes to explore the involved biological functions and pathways. According to KEGG analysis, these DEGs were primarily implicated in the PI3K‐Akt signaling pathway, extracellular matrix (ECM)–receptor interaction, and focal adhesion (Figure 3a). GO enrichment analysis displayed that in the category of BP, these DEGs were principally implicated in the terms of extracellular structure organization, ECM organization, and cell–substrate adhesion (Figure 3b). In the category of CC, these DEGs were mainly implicated in the ECM, collagen‐containing ECM, endoplasmic reticulum lumen, and contractile fiber (Figure 3c). In the category of MF, these DEGs were chiefly enriched in the glycosaminoglycan binding, growth factor binding, cytokine binding, and so on (Figure 3d). These pathways might exert an important role in the development of STAD.

Figure 3Functional enrichment analyses of the DEGs between two molecular subtypes. (a) KEGG enrichment analysis shown in the bubble plot. (b) GO enrichment analysis in biological process shown in the bubble plot. (c) GO enrichment analysis in cellular component shown in the bubble plot. (d) GO enrichment analysis in molecular function shown in the bubble plot.

**Figure:**
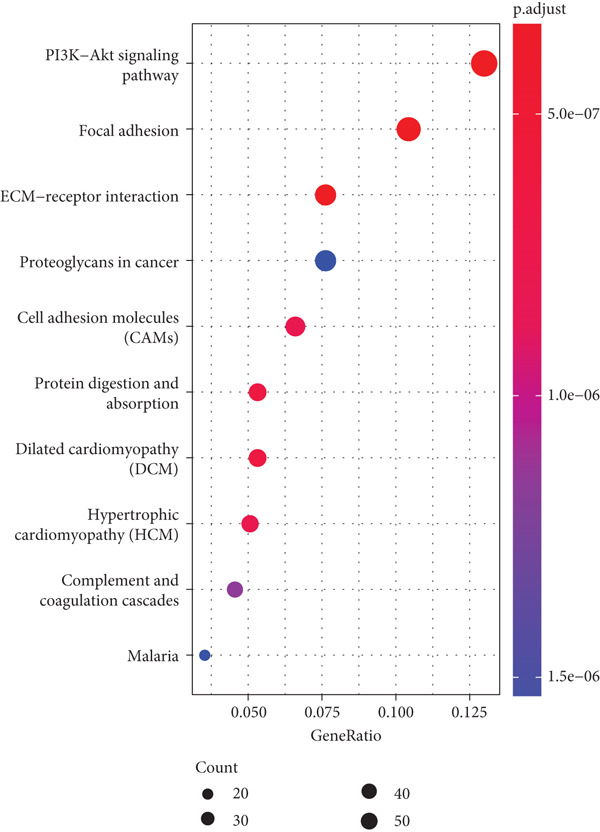
(a)

**Figure:**
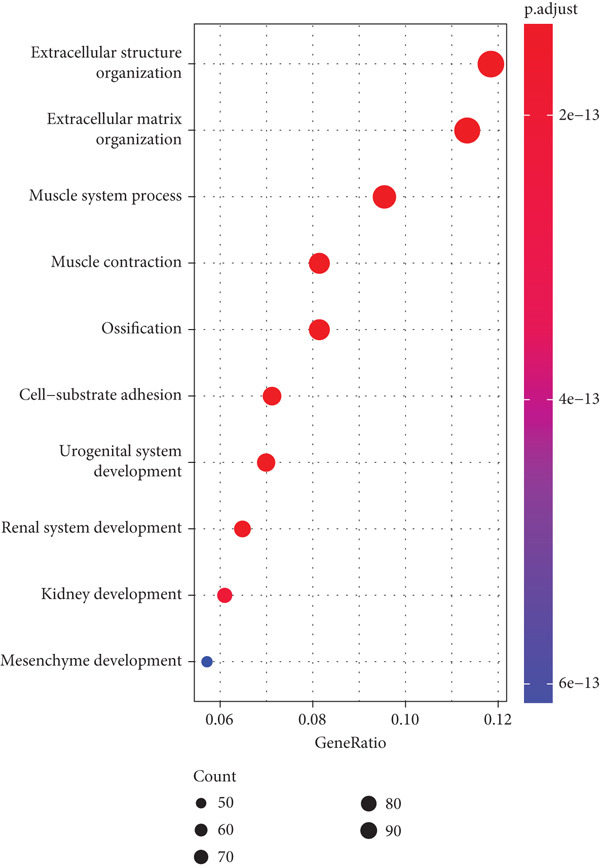
(b)

**Figure:**
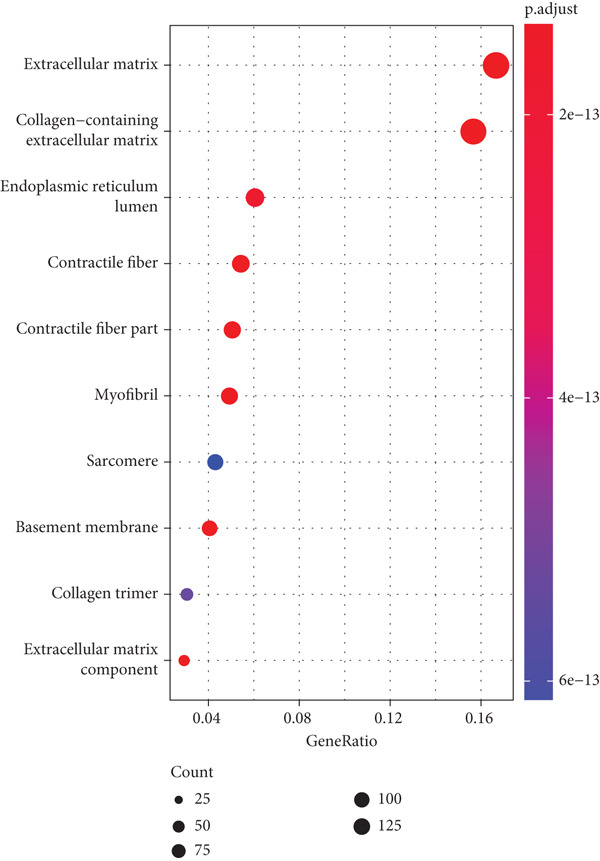
(c)

**Figure:**
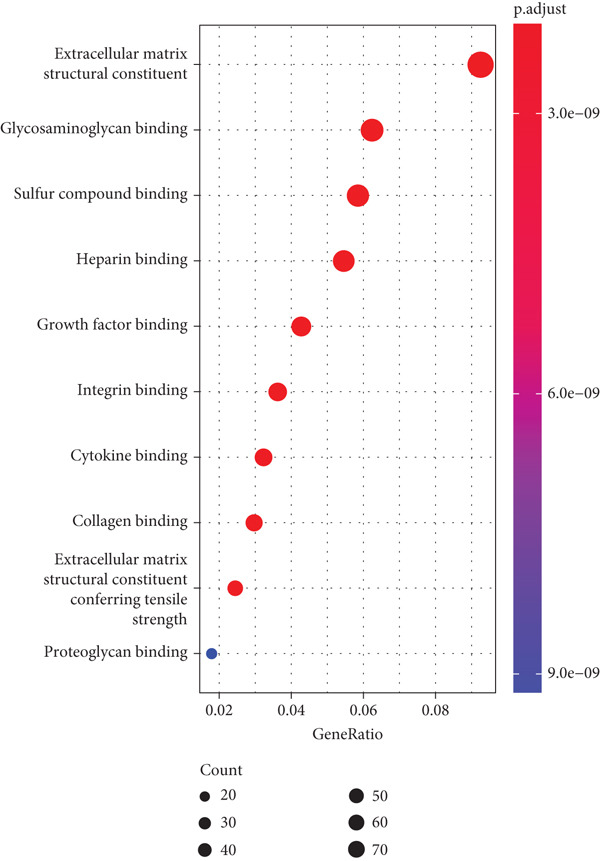
(d)

### 3.4. The RiskScore Model Was Created and Validated Based on Three Prognostic Signatures Related to PANoptosis

A sum of 382 prognostic genes was preliminarily screened from the 854 DEGs via univariate Cox regression analysis. Next, gene numbers in the model were compressed by LASSO Cox regression analysis. We found that when lambda = 0.0462, the model was optimal (Figure 4a,b). Further, through stepwise multivariate regression analysis, three prognostic gene signatures related to PANoptosis were identified, including *APOD*, *GPC3*, and *SERPINE1* (Figure 4c). Then, the RiskScore model was established: “RiskScore = 0.102∗APOD + 0.063∗GPC3 + 0.174∗SERPINE1.”

Figure 4Development of prognostic model and verification. (a) Coefficients of each variable. (b) Confidence intervals of each lambda. (c) Multivariate forest map of prognostic gene signatures;  ^∗∗^ *p* < 0.01 and  ^∗^ *p* < 0.05. (d) Expression of the signature genes, RiskScore, and survival status in the TCGA‐STAD cohort. (e) ROC curve of the RiskScore model and the K‐M curve of different risk groups in the TCGA‐STAD cohort. (f) RiskScore, survival status, and signature gene expression level in the GSE62254 dataset. (g) ROC curve of the RiskScore model and the K‐M curve of different risk groups in the GSE62254 dataset.

**Figure:**
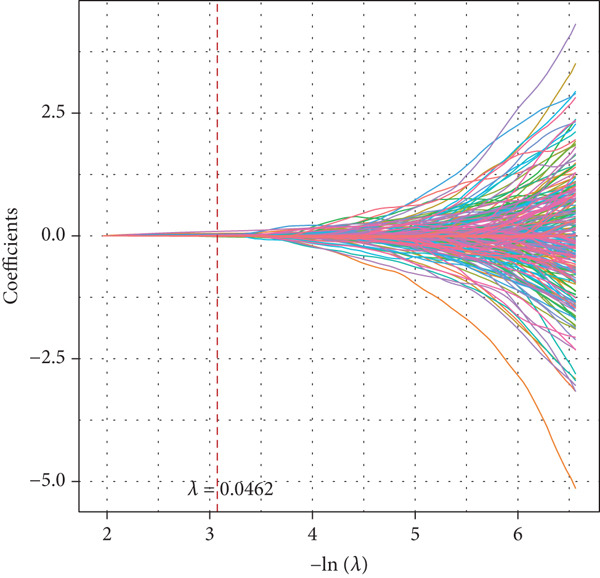
(a)

**Figure:**
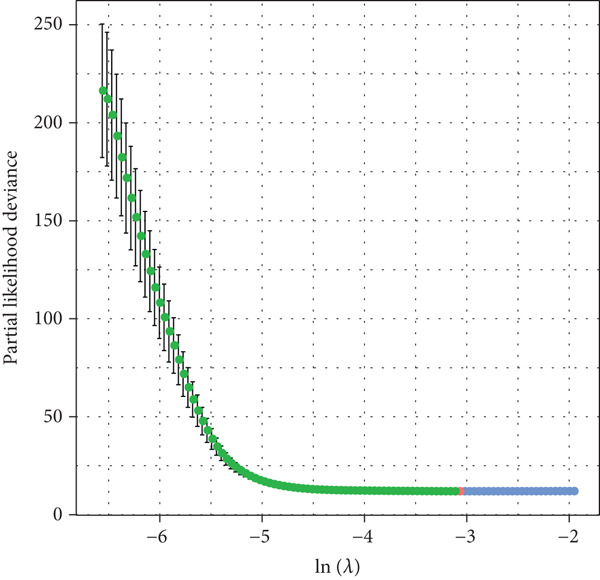
(b)

**Figure:**
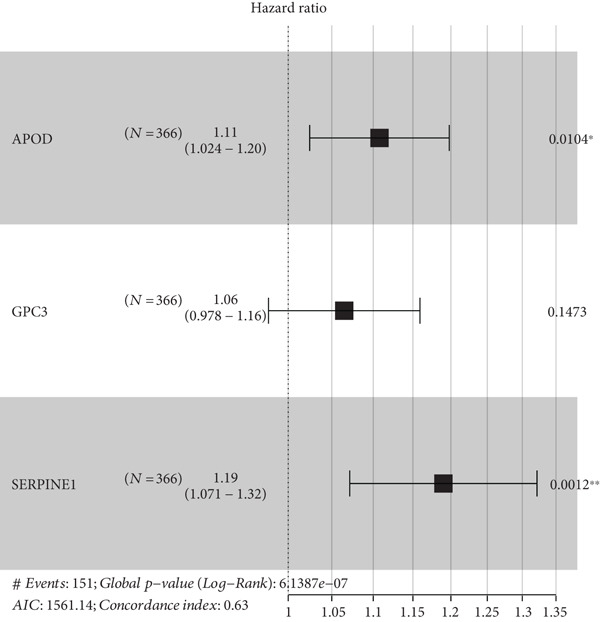
(c)

**Figure:**
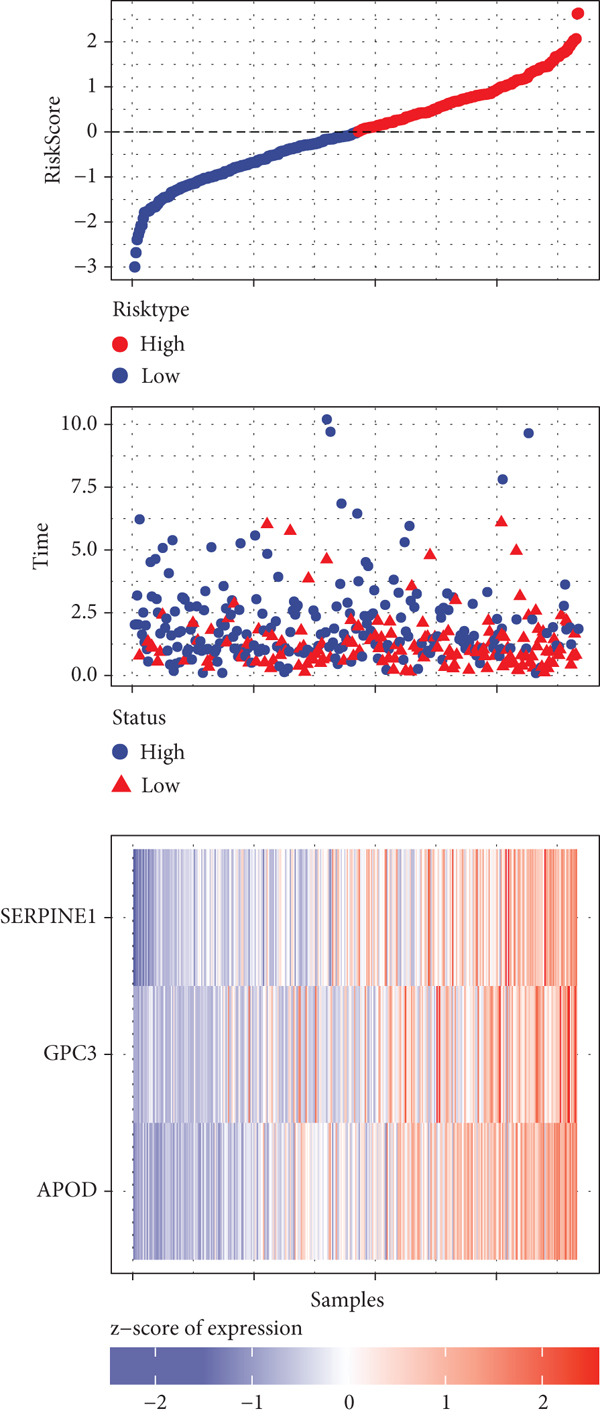
(d)

**Figure:**
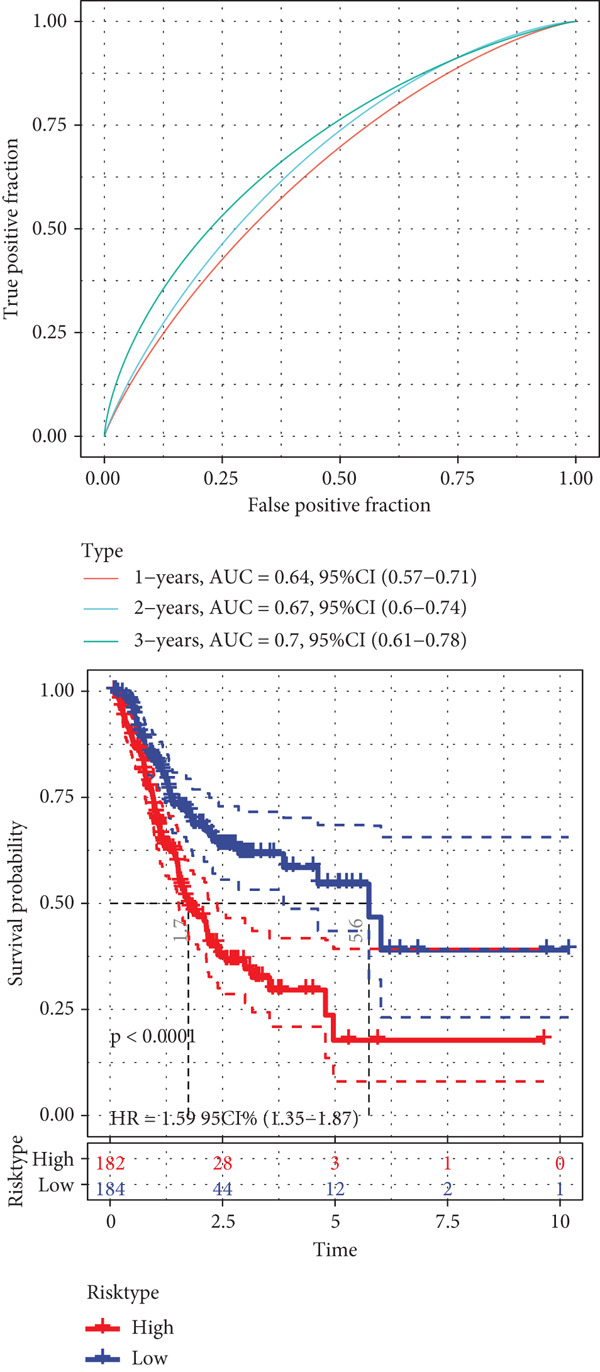
(e)

**Figure:**
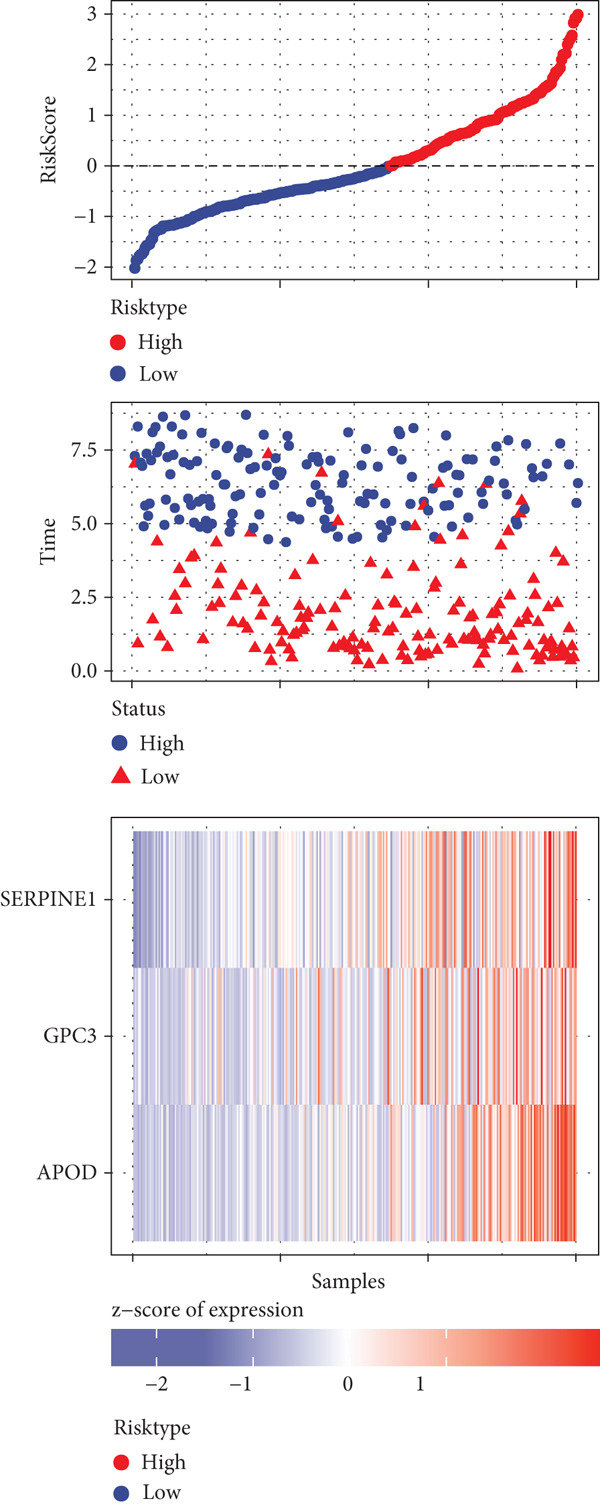
(f)

**Figure:**
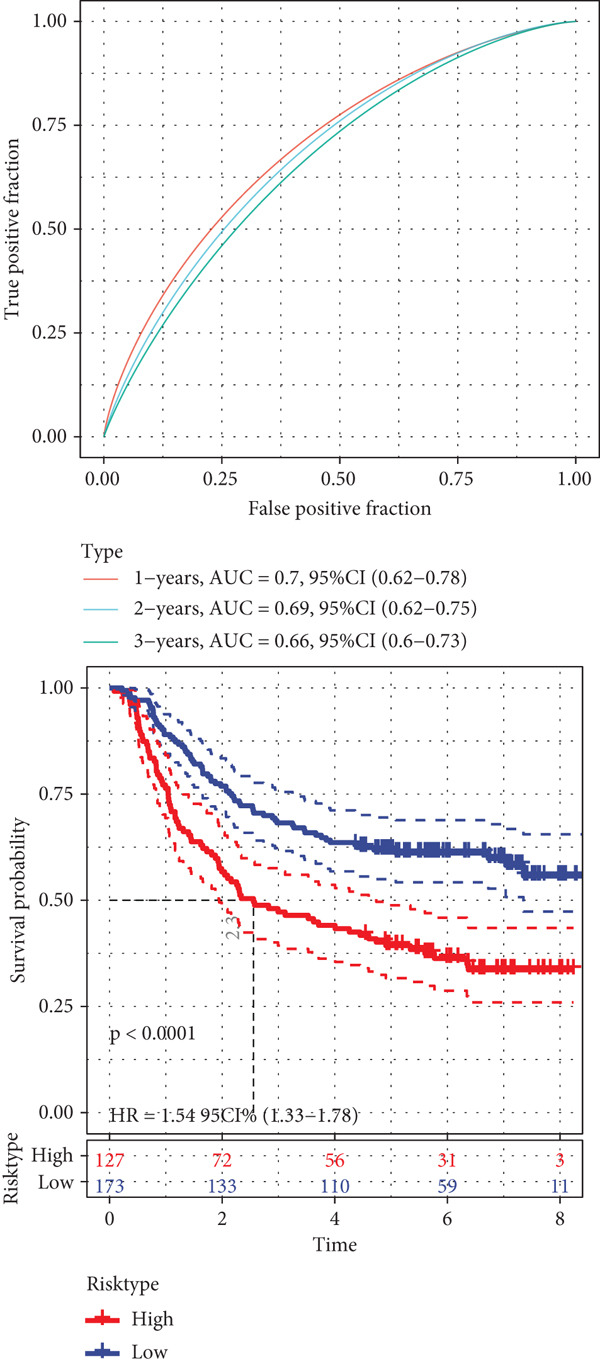
(g)

According to the cutoff value of RiskScore = 0, the high‐/low‐risk groups of patients in the TCGA‐STAD cohort were divided (Figure 4d). The RiskScore model had good predictive performance, with 1‐, 2‐, and 3‐year AUC of 0.64, 0.67, and 0.7 (Figure 4e). The high‐risk group showed a shorter OS in comparison with the low‐risk group (Figure 4e), indicating a poorer prognosis in the high‐risk STAD group. Besides, we validated the robustness of the RiskScore model in the GSE62254 dataset. The high‐/low‐risk groups of patients in GSE62254 dataset were also divided (Figure 4f), with the high‐risk group showing a lower OS rate, and the 1‐, 2‐, and 3‐year AUC values of the RiskScore model were 0.7, 0.69, and 0.66 (Figure 4g), which demonstrated that the RiskScore model demonstrated a strong potential in predicting STAD patient prognosis.

### 3.5. Development of a Nomogram Integrating RiskScore and Clinical Features and Validation

Whether the RiskScore was independent of other prognostic indicators was analyzed through univariate and multivariate Cox regression methods in the TCGA‐STAD cohort. We found that RiskScore was an independent prognostic factor for STAD (Figure 5a,b). Subsequently, a nomogram was developed combining clinical features and RiskScore (Figure 5c). The predicted calibration curve at the 1‐, 2‐, and 3‐year OS was close to the standard curve, and the DCA curve illustrated that the nomogram had a higher net benefit than other prognostic indicators (Figure 5d). These outcomes manifested that the nomogram was dependable in forecasting the prognosis of STAD patients.

Figure 5Establishment and validation of a nomogram. (a, b) The results of univariate and multivariate Cox regression analyses were visualized in forest plots. (c) Nomogram constructed with RiskScore and clinical features. (d) Calibration curve and DCA curve of the nomogram.  ^∗∗∗^ *p* < 0.001,  ^∗∗^ *p* < 0.01, and  ^∗^ *p* < 0.05.

**Figure:**
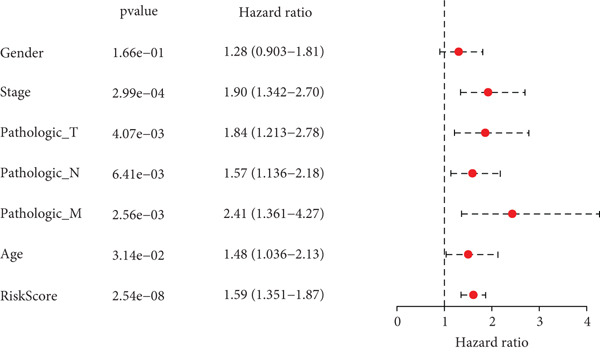
(a)

**Figure:**
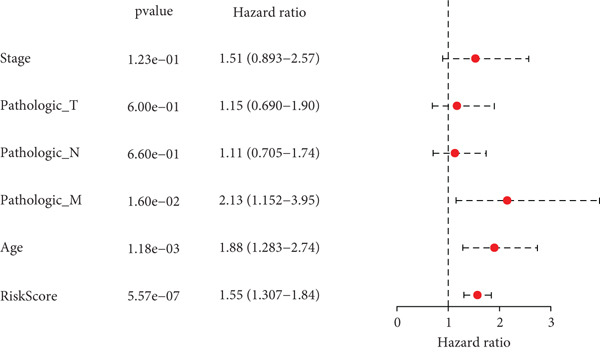
(b)

**Figure:**
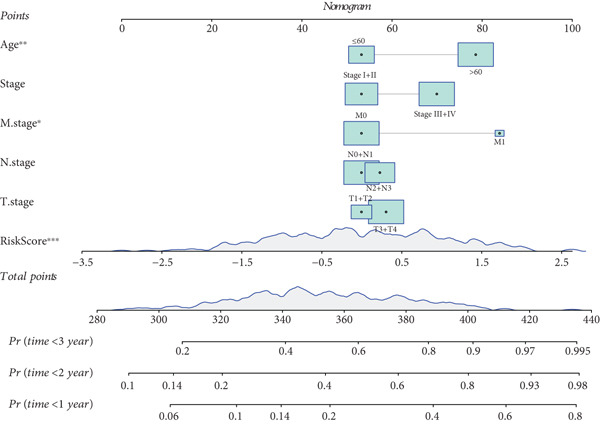
(c)

**Figure:**
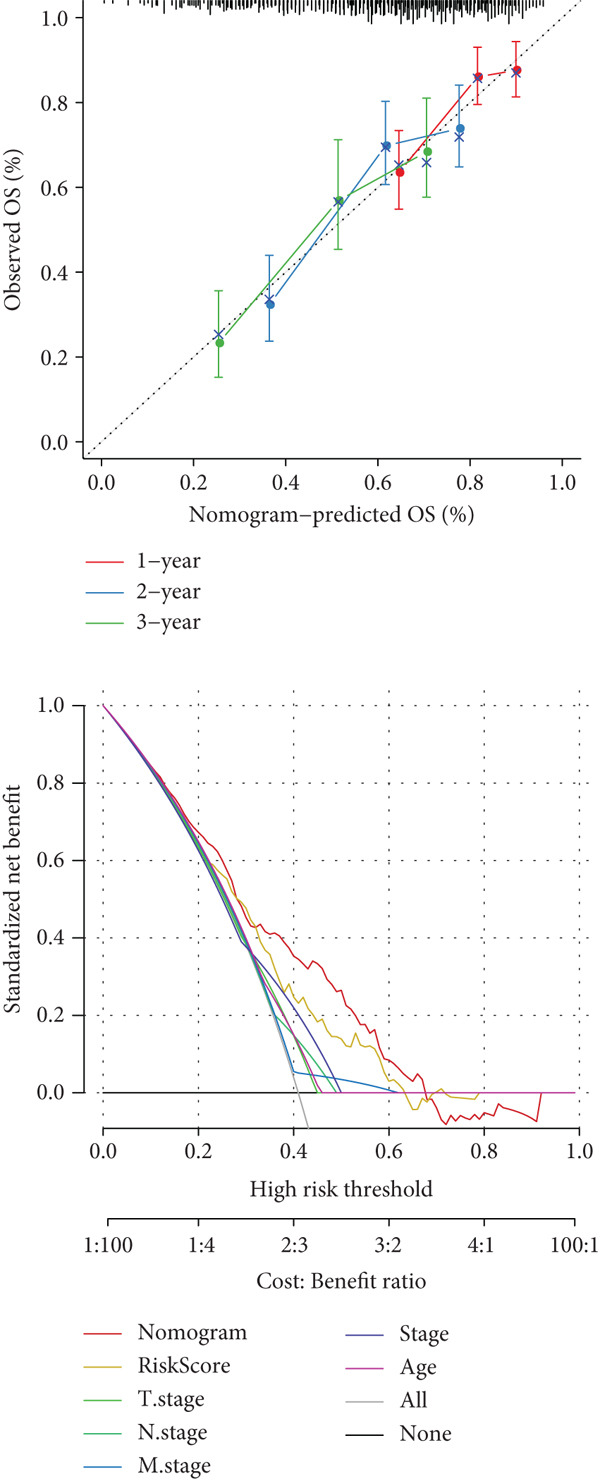
(d)

### 3.6. Correlation Between Immunotherapy Response, Drug Sensitivity, and RiskScore Was Analyzed

TIMER analysis displayed that the infiltration of macrophage, dendritic cell (DC), CD4 T cell, neutrophil, and CD8 T cell was markedly higher in the high‐risk group than the low‐risk group (Figure 6a). Compared to the low‐risk group, the MCP‐counter analysis suggested the high‐risk group with higher abundance of myeloid DC, cytotoxic lymphocytes, T cells, neutrophils, and B lineage (Figure 6b). Furthermore, the exclusion, dysfunction, and high‐risk group had notably higher TIDE scores in comparison to the low‐risk group (Figure 6c). RiskScore was positively related to nine immune checkpoint genes, like *CD244*, *CD27*, *IDO2*, *PDCD1, LAG3*, and *LAIR1* (Figure 6d). These results suggested that STAD patients with a higher RiskScore may have a stronger immune escape ability and benefit less from immunotherapy. Additionally, we screened 23 drugs with significant correlation with RiskScore (FDR < 0.05 and |cor| > 0.3), such as Erlotinib_1168, Gefitinib_1010, Lapatinib_1558, Ulixertinib_1908, BMS.754807_2171, JQ1_2172, AZD8055_1059, and NU7441_1038 (Figure 6e).

Figure 6Immunotherapy response prediction and drug sensitivity analysis. (a) TIMER method assessed the infiltration of six types of immune cells. (b) MCP‐counter algorithm quantified the abundance of 10 types of immune cells. (c) Dysfunction, exclusion, and TIDE scores of different risk groups. (d) Correlation between immune checkpoint genes and RiskScore. (e) Correlation between drugs IC_50_ and RiskScore;  ^∗^ *p* < 0.05,  ^∗∗^ *p* < 0.01,  ^∗∗∗^ *p* < 0.001, and  ^∗∗∗∗^ *p* < 0.0001.

**Figure:**
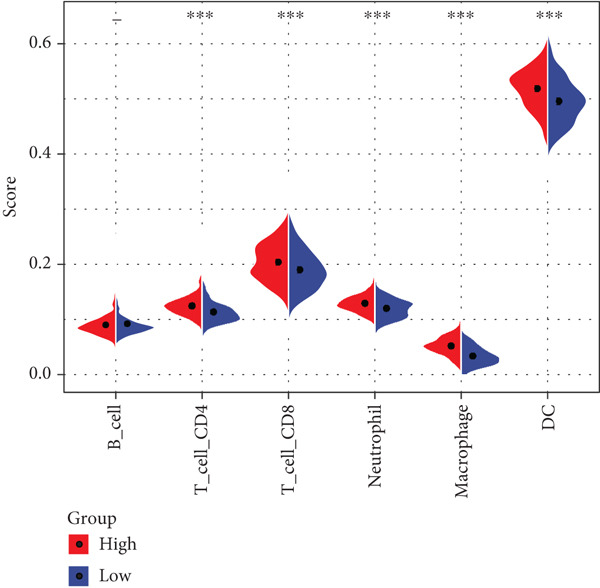
(a)

**Figure:**
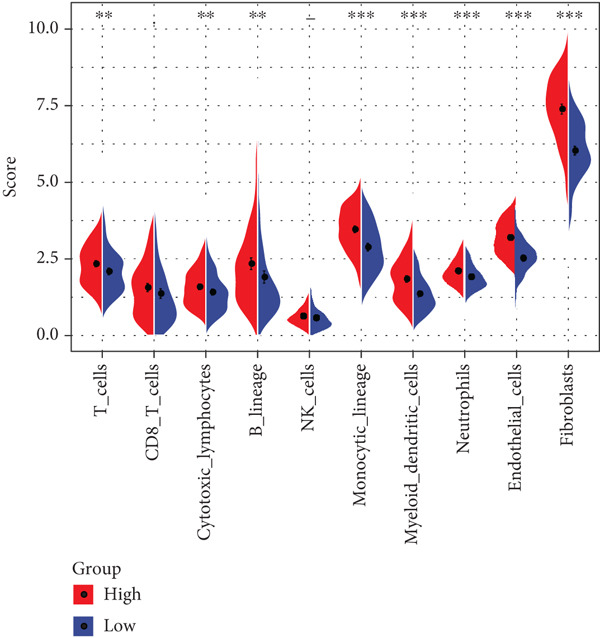
(b)

**Figure:**
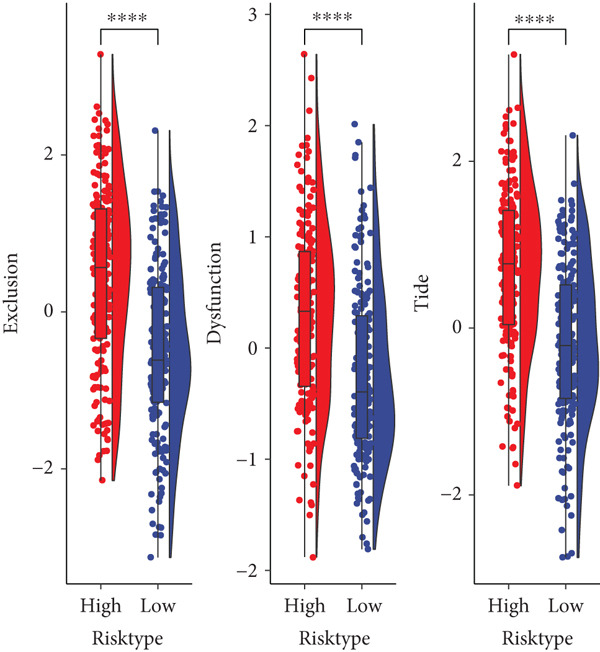
(c)

**Figure:**
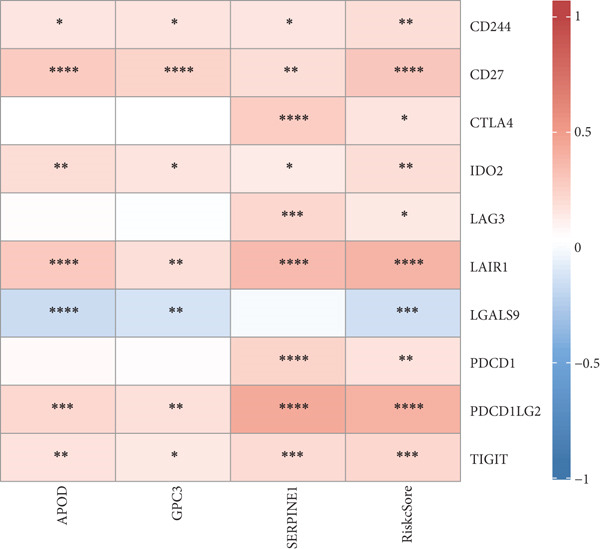
(d)

**Figure:**
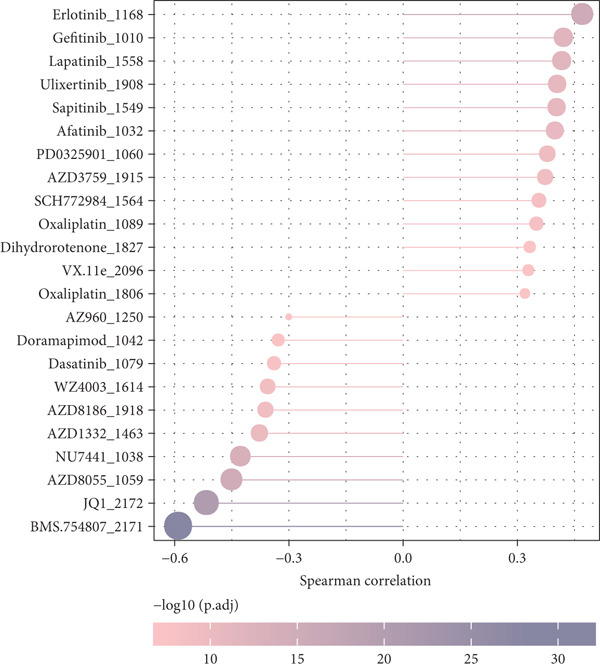
(e)

### 3.7. The Function of STAD Cells Was Impeded After Silencing *APOD*

The qRT‐PCR and Western blotting test showed the higher expression of *APOD*, *GPC3*, and *SERPINE1* in two STAD cell lines (AGS and HGC‐27) than those in the human stomach epithelial cell (GES‐1) (Figure 7a,b). Then, we selected *APOD* to silence, and the transfection efficiency was validated in STAD cells by the qRT‐PCR test (Figure 7c). The si‐APOD#1 with good knockdown efficiency was selected for subsequent verification. CCK‐8 test displayed that the cell viabilities of AGS and HGC‐27 cells were impaired after silencing *APOD* (Figure 7d). In addition, we observed that the migration and invasion abilities of APOD‐silenced STAD cells were impaired, while their apoptotic abilities were enhanced (Figures 7e, 7f, 7g, 7h, 7i, and 7j). These outcomes emphasized the potential roles of PRGs in STAD development.

Figure 7Roles of prognostic PRGs in STAD cell lines by in vitro experiments. (a) The qRT‐PCR detecting the expression of *APOD*, *GPC3*, and *SERPINE1* in human stomach epithelial cells (GES‐1) and STAD cells (AGS and HGC‐27). (b) Western blotting was used to assess the protein expression of *APOD*, *GPC3*, and *SERPINE1* in STAD cells. (c) The qRT‐PCR detecting the transfection efficiency of si‐APOD in two STAD cell lines. (d) CCK‐8 assay measuring the cell viability of *APOD*‐silenced HGC‐27 and AGS cells. (e–g) The effect of *APOD* gene silencing on the migration, invasion, and apoptosis abilities of AGS cells. (h–j) The effect of *APOD* gene silencing on the migration, invasion, and apoptosis abilities of HGC‐27 cells;  ^∗∗∗∗^ *p* < 0.0001,  ^∗∗∗^ *p* < 0.001,  ^∗∗^ *p* < 0.01, and  ^∗^ *p* < 0.05.

**Figure:**
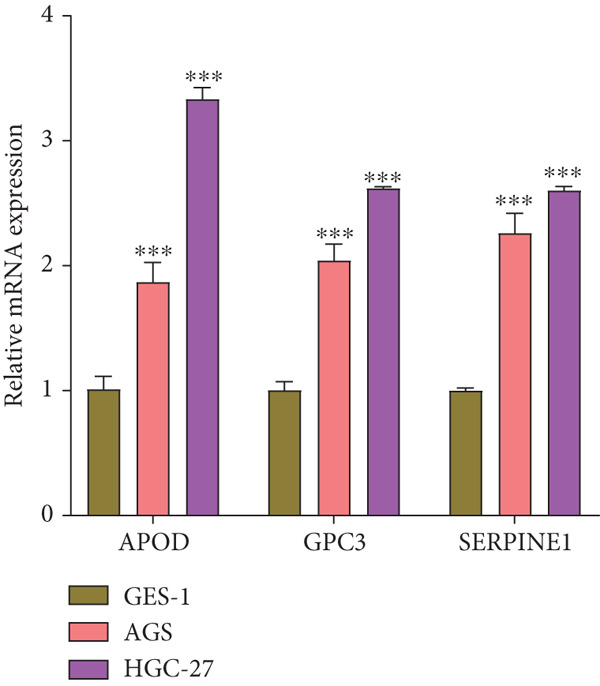
(a)

**Figure:**
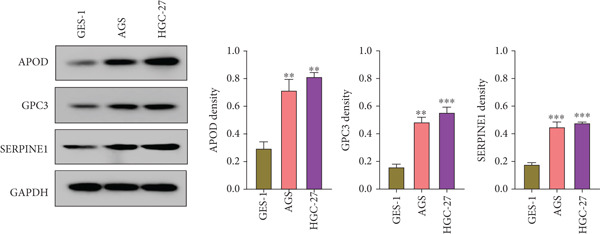
(b)

**Figure:**
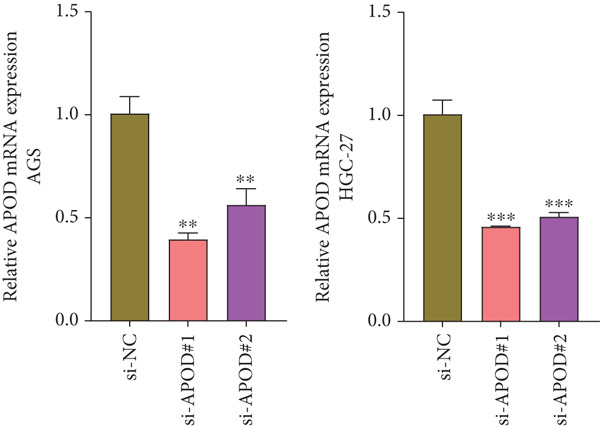
(c)

**Figure:**
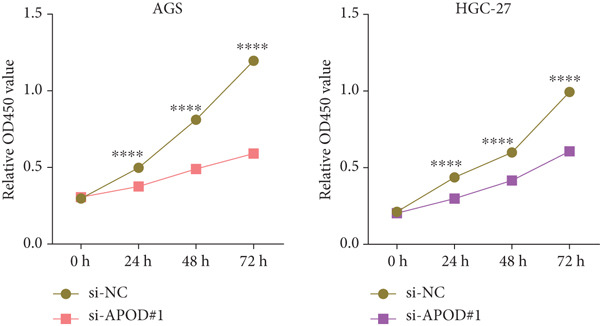
(d)

**Figure:**
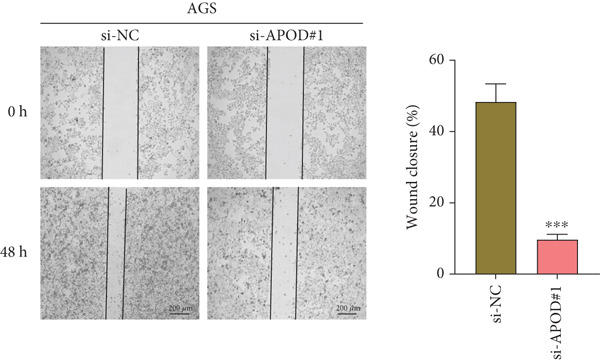
(e)

**Figure:**
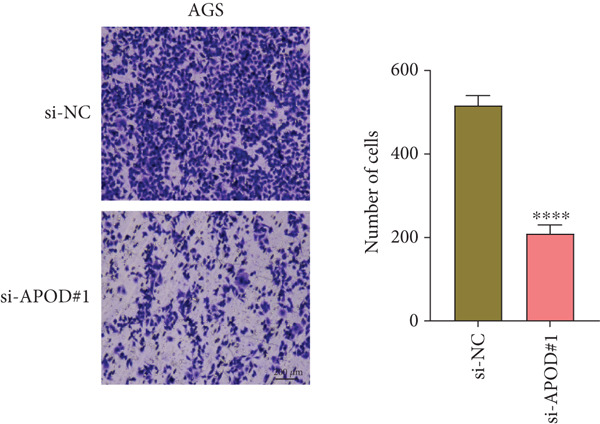
(f)

**Figure:**
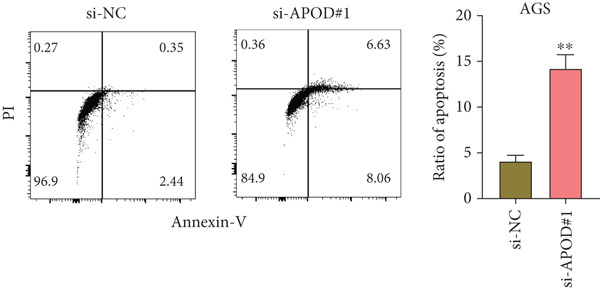
(g)

**Figure:**
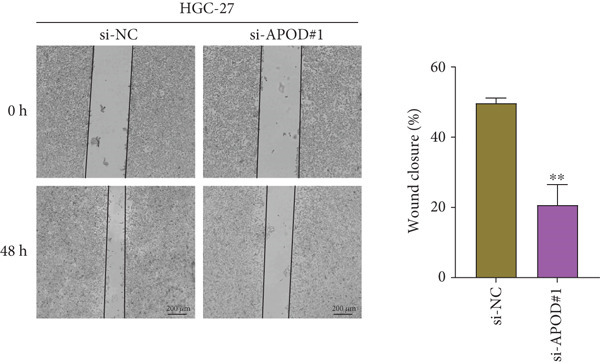
(h)

**Figure:**
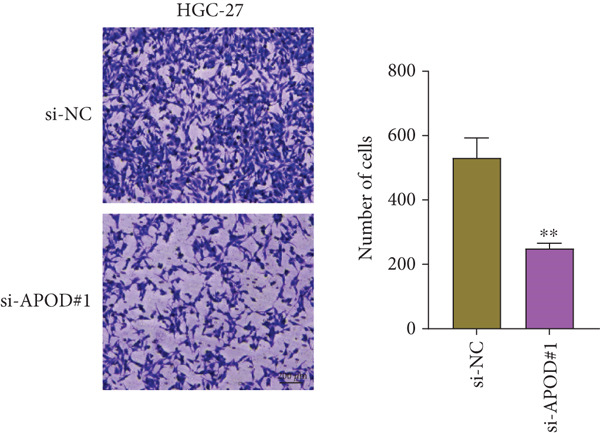
(i)

**Figure:**
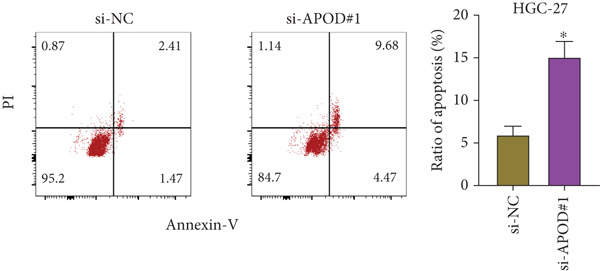
(j)

## 4. Discussion

STAD is a prevalent malignancy of the alimentary canal, and its prognostic indicators are critical for precise treatment [39]. PANoptosis, a novel form of inflammatory PCD discovered in 2019 by Malireddi et al. [40], has been manifested to be linked to multiple cancers, including GC [41]. For example, Pan et al. developed a PANscore system that could accurately evaluate the survival rate and immune efficacy of GC patients [42]. In this current study, based on 18 prognosis‐related PRGs, two molecular subtypes of STAD were recognized, and the C1 subtype had a lower OS rate and a higher clinical stage than the C2 subtype. Then, three PANoptosis‐related signatures (*APOD*, *GPC3*, and *SERPINE1*) were identified to develop a RiskScore model, which demonstrated strong performance in evaluating the immunotherapy response and prognostic outcome of STAD patients.

*APOD*, an apolipoprotein regulated via androgens and estrogens, can bind to arachidonic acid and regulate the oxidative stress and inflammatory response [43]. *APOD* has been identified as a part of the risk model in STAD and is relevant to tumor mutation burden and immune cell infiltration [44]. *GPC3*, belonging to the heparin sulfate glypican family, is anchored at the surface of the cell membrane via phosphatidylinositol [45]. *GPC3* acts as a critical function in the tumor proliferation, invasiveness, and deteriorative metastasis [46]. It has been reported that the *GPC3* expression GC accounts for about 11% of GC cases [47]. In addition, *SERPINE1*, an essential member of the serpin E superfamily, is also related to the progression of GC [48]. The upregulation of *SERPINE1* in GC tissues is conducive to the GC cell proliferation, invasion, and migration [49]. In STAD, Zhai et al. uncovered that the *SERPINE1* expression was enhanced with the advancement of tumor T, N, and M classification, and the high *SERPINE1* expression group had higher infiltration of immune cells compared with the low *SERPINE1* expression group [50]. In our study, *APOD*, *GPC3*, and *SERPINE1* were identified as the prognostic gene signatures linked to PANoptosis in STAD, based on which, RiskScore model was constructed with good robustness in predicting STAD patient prognosis. Besides, RiskScore was independent of other clinical features. In vitro assays indicated that *APOD*, *GPC3*, and *SERPINE1* were upregulated in STAD cells. Silencing the expression of *APOD* significantly impaired the cell functions of STAD. These findings manifested that the PRGs could serve as reliable prognostic predictors for STAD and might become potential therapeutic targets.

Past studies reported that TME also acts as a key role in the progression, prognosis, and immunotherapy response of STAD [51]. Elaborating on the features of TME in STAD could help us further understand the pathogenesis of STAD and offer some references for personalized treatment [52]. In contrast to the low‐risk group, the current study found higher infiltration of immune cells like cytotoxic lymphocytes, B lineage, CD8 T cells, neutrophils, CD4 T cells, macrophages, and DC in the high‐risk group. Moreover, the TIDE score of the high‐risk group was notably higher, indicating that high‐risk STAD patients may benefit less from immunotherapy [53]. We also found that RiskScore was positively linked to nine immune checkpoint genes, comprising *CD244*, *CD27*, *CTLA4*, *IDO2*, *LAG3*, *LAIR1*, *PDCD1*, *PDCD1LG2*, and *TIGIT*, which demonstrated that STAD patients with higher RiskScore may have stronger immune evasion ability [54]. Based on these findings, we speculated that STAD patients in the high‐risk group, despite high immune cell infiltration, may have strong immune escape capacity that is closely linked to their poor prognosis. In addition, 23 drugs that showed significant correlation with RiskScore were screened in this study, such as Erlotinib_1168, Gefitinib_1010, Lapatinib_1558, and Ulixertinib_1908. Erlotinib is approved as the first‐line therapy drug for advanced non–small cell lung cancer [55] and pancreatic cancer [56]. Lapatinib, a small molecule tyrosine kinase inhibitor, shows antitumor effect on GC cell lines [57]. Ulixertinib, a first‐in‐class ERK inhibitor, is an oral drug with a safety profile and has preclinical anticancer activity in vitro and in vivo [58]. Gefitinib can exert an antitumor activity in GC by suppressing epidermal growth factor receptor; however, its efficacy is limited due to drug resistance [59]. Therefore, it is necessary to further explore the specific efficacy and resistance mechanism of these drugs in STAD to provide insights for developing new STAD treatment strategies.

Nonetheless, some limitations in the current work should be equally noted. First, this study primarily used only two publicly available datasets and lacked large‐scale validation across multiple centers, ethnicities, and sequencing platforms. The model′s generalizability and robustness still require further confirmation. In the future, we will introduce more independent, multicenter STAD cohorts (including data from different ethnicities and testing platforms) and combine them with prospectively collected clinical samples for external validation to enhance the model′s applicability and clinical value. Second, there is a significant imbalance between the number of tumor samples and adjacent normal samples in the TCGA data. Although the main analyses were performed within the tumor samples and the comparison of normal samples was only used for background display, this imbalance may introduce bias in some differentially expressed results. In addition, this study did not include GTEx normal gastric tissue data. Therefore, in future studies, we will attempt to incorporate GTEx normal samples that have undergone rigorous batch effect correction or supplement the normal control group with multicenter clinical tissue samples to further enhance the robustness of differential analysis. Third, the RiskScore model constructed in this study is based on RNA‐seq data, but the external validation set GSE62254 is chip detection data. Although it has undergone rigorous standardization and normalization and the validation results show that the model performs well on both platforms, technical platform differences may affect prediction performance. We will further validate the model across more RNA‐seq platform cohorts and datasets with varying sequencing depths and quality control standards to comprehensively assess its stability and applicability under diverse technical conditions. Fourth, the predictive ability of the model in this study was moderate in the training and validation sets. Although it demonstrated cross‐platform stability and independent prognostic value, there is still room for improvement. In future studies, we will consider introducing more molecular features closely related to PANoptosis and STAD prognosis, combining clinical parameters and multiomics data to optimize the model structure and improve predictive performance and clinical applicability. Fifth, this study only validated the expression and function of core genes at the cellular level, but there is still a lack of mRNA and protein level detection in clinical tissue samples. In the future, we will supplement the collection of STAD patient tissue samples, validate the expression of core genes at the tissue level, and combine patient prognosis data for correlation analysis to enhance the clinical relevance of the research conclusions. Finally, the current functional experiments have only been validated by knockdown of the APOD gene, which is insufficient evidence of the “sufficiency” of gene action. Therefore, subsequent overexpression experiments will be conducted, accompanied by knockdown‐rescue experiments, while detecting PANoptosis‐related molecular markers to comprehensively reveal the bidirectional regulatory role of core genes in the PANoptosis process.

## 5. Conclusion

In summary, we revealed two molecular subtypes of STAD and three PANoptosis‐related signatures (*APOD*, *GPC3*, and *SERPINE1*), which were utilized to construct the RiskScore model with good robustness in assessing the immunotherapy response and prognostic outcomes for STAD patients. Moreover, RiskScore was independent of other clinical features. This study could provide some references for prognosis assessment and personalized treatment of STAD patients.

**Nomenclature**
ANOVA: analysis of variance
AUC: area under the ROC curve
BP: biological process
CC: cellular component
CCK‐8: Cell Counting Kit‐8
DC: dendritic cell
DCA: decision curve analysis
DEGs: differentially expressed genes
ECM: extracellular matrix
FBS: fetal bovine serum
FC: foldchange
FDR: false discovery rate
FPKM: fragments per kilobase per million
GC: gastric cancer
GEO: Gene Expression Omnibus
GO: Gene Ontology
HR: hazard ratio
IC50: half maximum inhibitory concentration
KEGG: Kyoto Encyclopedia of Genes and Genomes
K‐M: Kaplan–Meier
LASSO: least absolute shrinkage and selection operator
MCP‐counter: microenvironment cell population‐counter
MF: molecular function
OS: overall survival
PCD: programmed cell death
PRGs: PANoptosis‐related genes
qRT‐PCR: quantitative reverse transcription PCR
ROC: receiver operating characteristic
si: small interfering
ssGSEA: single sample gene set enrichment analysis
STAD: stomach adenocarcinoma
TCGA: The Cancer Genome Atlas
TIDE: Tumor Immune Dysfunction and Exclusion
TIMER: Tumor Immune Estimation Resource
TME: tumor microenvironment
TPM: transcripts per million
UCSC Xena: University of California, Santa Cruz Xena

## Ethics Statement

The authors have nothing to report.

## Consent

The authors have nothing to report.

## Disclosure

All the authors have read and approved the manuscript.

## Conflicts of Interest

The authors declare no conflicts of interest.

## Author Contributions

All authors contributed to the present work: X.W. designed the study, and L.S. acquired the data. P.L., Q.W., and Y.W. improved the figure quality. X.W., L.S., and Y.W. drafted the manuscript. X.W., Q.W., and P.L. revised the manuscript.

## Funding

No funding was received for this manuscript.

## Data Availability

The datasets generated and/or analyzed during the current study are available in the GSE62254 repository, [https://www.ncbi.nlm.nih.gov/geo/query/acc.cgi?acc= GSE62254].
